# High-throughput sequencing SELEX for the determination of DNA-binding protein specificities *in vitro*

**DOI:** 10.1016/j.xpro.2022.101490

**Published:** 2022-06-23

**Authors:** Raphaël Pantier, Kashyap Chhatbar, Grace Alston, Heng Yang Lee, Adrian Bird

**Affiliations:** 1 The Wellcome Centre for Cell Biology, University of Edinburgh, Michael Swann Building, Max Born Crescent, The King’s Buildings, Edinburgh EH9 3BF, UK; 2 Informatics Forum, School of Informatics, University of Edinburgh, 10 Crichton Street, Edinburgh EH8 9AB, UK

**Keywords:** Bioinformatics, Sequence analysis, Sequencing, High Throughput Screening, Molecular Biology

## Abstract

High-throughput sequencing SELEX (HT-SELEX) is a powerful technique for unbiased determination of preferred target motifs of DNA-binding proteins *in vitro*. The procedure depends upon selection of DNA binding sites from a random library of oligonucleotides by purifying protein-DNA complexes and amplifying bound DNA using the polymerase chain reaction. Here, we describe an optimized step-by-step protocol for HT-SELEX compatible with Illumina sequencing. We also introduce a bioinformatic pipeline (eme_selex) facilitating the detection of promiscuous DNA binding by analyzing the enrichment of all possible k-mers.

For complete details on the use and execution of this protocol, please refer to Pantier et al. (2021).

## Highlights

• HT-SELEX requires a random DNA library and recombinant DNA-binding proteins
• HT-SELEX identifies preferred sequence motifs of DNA-binding proteins
• eme_selex quantifies all possible k-mers to detect promiscuous DNA binding

## Abstract (editor highlights)

Publisher’s note: Undertaking any experimental protocol requires adherence to local institutional guidelines for laboratory safety and ethics.

## Graphical abstract

**Figure:**
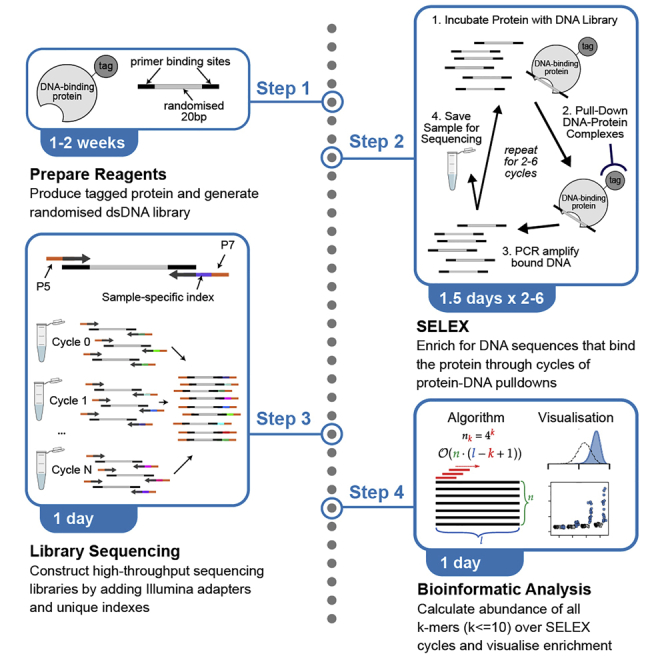

## Before you begin

Systematic evolution of ligands by exponential enrichment (SELEX) is a molecular biology technique allowing the *in vitro* selection of DNA oligonucleotide duplexes with high affinity for a target ligand (Ellington and Szostak, 1990; Tuerk and Gold, 1990). This technology can be coupled with high-throughput sequencing (HT-SELEX) to determine transcription factor binding specificities (Roulet et al., 2002; Jolma et al., 2010; Slattery et al., 2011).

Here, we describe the stepwise performance and analysis of HT-SELEX using purified SALL4 C2H2 zinc-finger clusters as “bait” (Pantier et al., 2021). However, this protocol can be applied to a wide range of DNA-binding proteins or DNA-binding domains (see limitations). Two critical reagents are required to initiate HT-SELEX: a library of random oligonucleotides; and a purified DNA-binding protein fused with an affinity tag.

### Generate a random library of double-stranded DNA oligonucleotides (cycle 0)

**Timing: 1 day**

**CRITICAL:** PCR conditions were optimized to amplify SELEX libraries. However, the amount of DNA template and the number of PCR cycles might need to be adjusted in order to avoid the formation of heteroduplexes (see troubleshooting 1).

**Figure 1:**
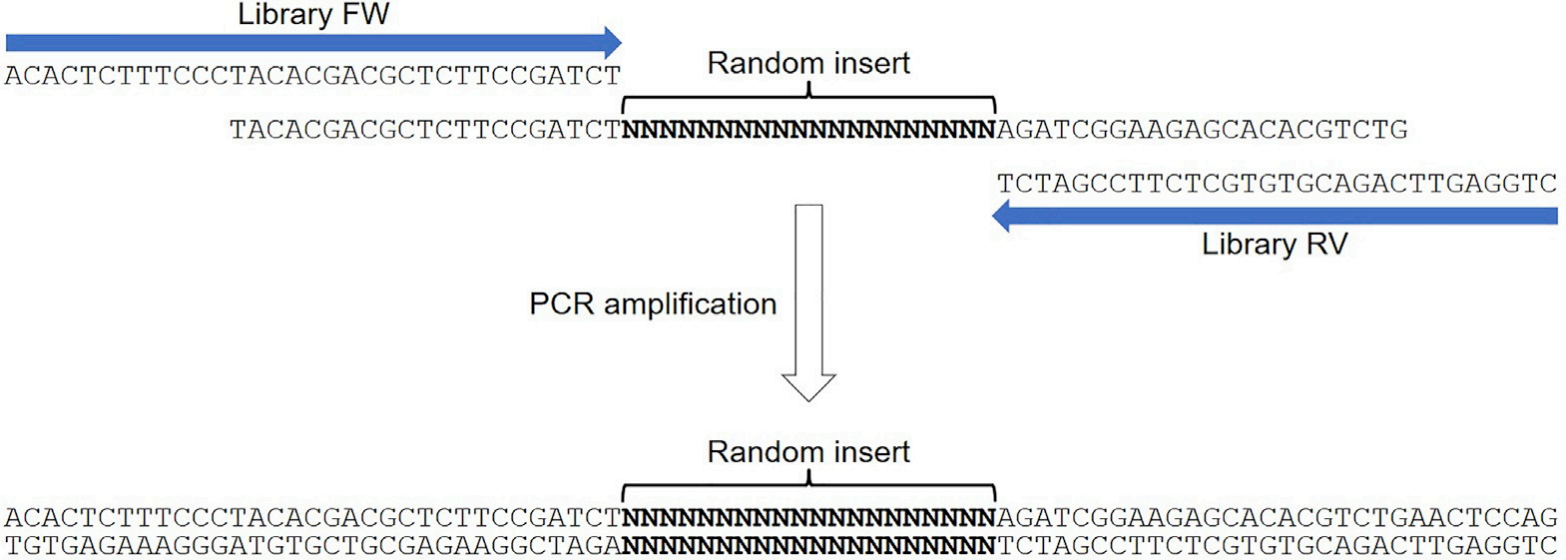
Strategy for generating a double-stranded DNA library using a single-stranded DNA template and two flanking primers The initial SELEX library (cycle 0) contains a “random insert”, corresponding to all putative sequence motifs for DNA-binding proteins.

***Note:*** Three separate random oligonucleotide libraries are used as technical replicates within the SELEX protocol, given that each library will present a slightly different distribution of DNA sequences.

***Note:*** A relatively large amount of random library DNA is necessary to initiate the SELEX protocol (1.5 μg library/sample for the first cycle). Each PCR reaction yields around 500 ng of DNA, so 24× PCR will generate enough material for 8× SELEX samples (≈12 μg DNA). Scale up or down as necessary.

1. For each DNA template (Random library 1/2/3, see materials and equipment), prepare a PCR mastermix (for 24× PCR reactions) in a 1.5 mL tube (Figure 1).

**Table:** PCR reaction master mix 1

Reagent	Amount
**“Random library 1” DNA template**	12 pmol (0.5 pmol/reaction) **Might be adjusted**
5× Phusion HF Buffer	240 μL
dNTPs (10 mM)	24 μL
Library FW (10 μM)	60 μL
Library RV (10 μM)	60 μL
Phusion DNA Polymerase	12 μL
Nuclease-free water	up to 1.2 mL
**Total**	**1.2 mL**

**Table:** PCR reaction master mix 2

Reagent	Amount
**“Random library 2” DNA template**	12 pmol (0.5 pmol/reaction) **Might be adjusted**
5× Phusion HF Buffer	240 μL
dNTPs (10 mM)	24 μL
Library FW (10 μM)	60 μL
Library RV (10 μM)	60 μL
Phusion DNA Polymerase	12 μL
Nuclease-free water	up to 1.2 mL
**Total**	**1.2 mL**

**Table:** PCR reaction master mix 3

Reagent	Amount
**“Random library 3” DNA template**	12 pmol (0.5 pmol/reaction) **Might be adjusted**
5× Phusion HF Buffer	240 μL
dNTPs (10 mM)	24 μL
Library FW (10 μM)	60 μL
Library RV (10 μM)	60 μL
Phusion DNA Polymerase	12 μL
Nuclease-free water	up to 1.2 mL
**Total**	**1.2 mL**

2. Divide each mastermix between 24 PCR tubes (50 μL/tube).
3. Run the following PCR programme:

**Table:** PCR cycling conditions

Steps	Temperature	Time	Cycles
Initial Denaturation	98°C	1 min	1
Denaturation	98°C	20 s	1 (initial PCR step)
Annealing	60°C	20 s	
Extension	72°C	20 s	
Denaturation	98°C	20 s	5 cycles
Annealing	68°C	20 s	**Might be adjusted**
Extension	72°C	20 s	
Final extension	72°C	5 min	1
Hold	4°C	forever

4. To verify the generation of double-stranded DNA libraries, run a small amount of PCR reaction (5 μL) on a 10% polyacrylamide gel and stain with a 0.5 μg/mL ethidium bromide solution. You should observe a single band at 83 bp and no detectable heteroduplexes (see Figure 2).

**Figure 2:**
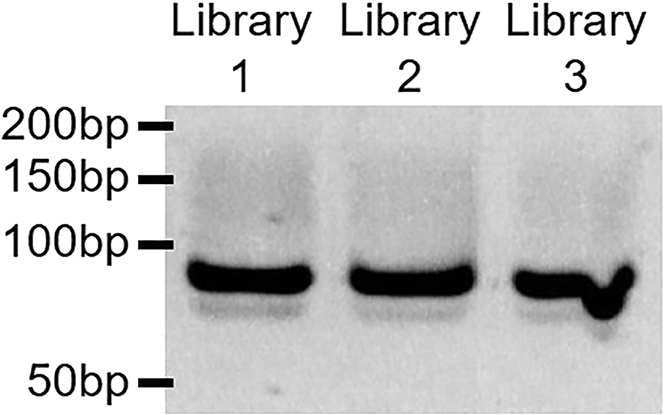
10% polyacrylamide gel showing the generation of three independent libraries of random oligonucleotides (cycle 0) to initiate the HT-SELEX protocol
5. Purify SELEX libraries using the Qiagen MinElute PCR purification kit and following manufacturer’s protocol. To obtain high concentrations, pool 8× identical PCR reactions into 1× MinElute column and elute with 20 μL of EB Buffer (included in the kit, 10 mM Tris-HCl pH8.5) or H_2_O.
6. Evaluate DNA concentration and integrity of purified SELEX libraries using a Nanodrop spectrophotometer.

### Purify DNA-binding proteins fused with an affinity tag

**Timing: 1–2 weeks**

Here, we describe the HT-SELEX protocol using histidine-tagged SALL4 C2H2 zinc-finger cluster 4 (ZFC4). We do not provide a generic protocol for protein expression and purification, as this process should be optimized for each individual protein. Detailed information regarding the choice of expression systems and purification strategies is extensively discussed in the literature (Gräslund et al., 2008; Kielkopf et al., 2021).

For more information regarding the purification of SALL4 ZFC4, please refer to our previously published manuscript (Pantier et al., 2021). Recombinant proteins were diluted to a concentration of 0.5 mg/mL in protein buffer (20 mM Tris-HCl pH7.5, 150 mM NaCl), and aliquots were stored at −80°C.

***Note:*** The addition of an affinity tag is critical both for purifying proteins from bacterial extracts and for purifying protein-DNA complexes during the SELEX protocol. We prefer the **hexahistidine tag** as it is small (6 residues) and allows for cost-efficient purification by immobilized metal affinity chromatography (IMAC). Other tags can be used to facilitate protein expression and solubilization (e.g., GST, MBP), but their larger size might impact the DNA binding capacity of fusion proteins.

## Key resources table

**Table:** 

REAGENT or RESOURCE	SOURCE	IDENTIFIER
**Chemicals, peptides, and recombinant proteins**		

(His)_6_-SALL4 ZFC4 recombinant protein	(Pantier et al., 2021)	N/A
Poly(dI-dC)	Merck Life Science	Cat#P4929-10UN
dNTPs	New England Biolabs (NEB)	Cat#N0447S
Ultrapure Tris Buffer	Thermo Fisher Scientific	Cat#15504020
HCl	Fisher Scientific	Cat#10316380
NaCl	Merck Life Science	Cat#71380-5KG
MgCl_2_	Merck Life Science	Cat#M9272-500G
DTT	Merck Life Science	Cat#D9779-1G
EDTA solution, 0.5 M	Merck Life Science	Cat#03690-100ML
Glycerol	Fisher Scientific	Cat#10336040
30% Acrylamide/Bis Solution, 37.5:1	Bio-Rad	Cat#1610158
TEMED	Merck Life Science	Cat#T9281-25ML
Ammonium persulfate	Merck Life Science	Cat#215589-100G
Ethidium bromide solution, 10 mg/mL	Merck Life Science	Cat#E1510-10ML
50 bp DNA ladder	New England Biolabs (NEB)	Cat#N3236S
Nuclease-Free Water	Thermo Fisher Scientific	Cat#AM9937
10× TBE buffer	Bio-Rad	Cat#1610770

**Critical commercial assays**		

Phusion DNA Polymerase	New England Biolabs (NEB)	Cat#M0530L
MinElute PCR Purification Kit	QIAGEN	Cat#28004
High Sensitivity DNA Kit	Agilent	Cat#5067-4626
KAPA Pure Beads	Roche	Cat#07983271001
Ni Sepharose 6 Fast Flow	Cytiva	Cat#17531806

**Deposited data**		

HT-SELEX of SALL4 C2H2 zinc-finger clusters	Array Express (https://www.ebi.ac.uk/arrayexpress/)	E-MTAB-9236 (Pantier et al., 2021), E-MTAB-11484 (This paper)

**Oligonucleotides**		

Random library 1	Integrated DNA Technologies (IDT)	N/A
Random library 2	Integrated DNA Technologies (IDT)	N/A
Random library 3	Integrated DNA Technologies (IDT)	N/A
Library FW	Integrated DNA Technologies (IDT)	N/A
Library RV	Integrated DNA Technologies (IDT)	N/A
Seqlib FW	Integrated DNA Technologies (IDT)	N/A
Seqlib RV1	Integrated DNA Technologies (IDT)	N/A
Seqlib RV2	Integrated DNA Technologies (IDT)	N/A
Seqlib RV3	Integrated DNA Technologies (IDT)	N/A
Seqlib RV4	Integrated DNA Technologies (IDT)	N/A
Seqlib RV5	Integrated DNA Technologies (IDT)	N/A
Seqlib RV6	Integrated DNA Technologies (IDT)	N/A
Seqlib RV7	Integrated DNA Technologies (IDT)	N/A
Seqlib RV8	Integrated DNA Technologies (IDT)	N/A
Seqlib RV9	Integrated DNA Technologies (IDT)	N/A
Seqlib RV10	Integrated DNA Technologies (IDT)	N/A
Seqlib RV11	Integrated DNA Technologies (IDT)	N/A
Seqlib RV12	Integrated DNA Technologies (IDT)	N/A
Seqlib RV13	Integrated DNA Technologies (IDT)	N/A
Seqlib RV14	Integrated DNA Technologies (IDT)	N/A
Seqlib RV15	Integrated DNA Technologies (IDT)	N/A
Seqlib RV16	Integrated DNA Technologies (IDT)	N/A
Seqlib RV17	Integrated DNA Technologies (IDT)	N/A
Seqlib RV18	Integrated DNA Technologies (IDT)	N/A
Seqlib RV19	Integrated DNA Technologies (IDT)	N/A
Seqlib RV20	Integrated DNA Technologies (IDT)	N/A
Seqlib RV21	Integrated DNA Technologies (IDT)	N/A

**Recombinant DNA**		

pET-(His)_6_-SALL4 ZFC4 expression plasmid	(Pantier et al., 2021)	N/A

**Software and algorithms**		

Every Motif Ever (eme_selex)	https://github.com/kashyapchhatbar/eme_selex (This paper)	https://doi.org/10.5281/zenodo.6586738
Flexbar 3.5.0	https://github.com/seqan/flexbar	N/A
Snakemake	https://github.com/snakemake/snakemake	N/A
Jupyterlab	https://jupyter.org/install	N/A
Pandas	https://pandas.pydata.org	N/A
Seaborn	https://seaborn.pydata.org	N/A

**Other**		

1.5 mL DNA LoBind tubes	Eppendorf	Cat#0030108051
Snapstrip II PCR tubes	Camlab	Cat#1147982
Mini-PROTEAN electrophoresis system	Bio-Rad	Cat#1658000
Electrophoresis Power Supply	Bio-Rad	Cat#1645050
2100 Bioanalyzer Instrument	Agilent	Cat#G2939BA
NanoDrop Spectrophotometer	Thermo Fisher Scientific	Cat#ND-1000
Alpha Cycler 4 PCR machine	PCRmax	Cat#AC496

## Materials and equipment

• Alternative choices of reagents.

***Alternatives:*** Here, we used the Phusion DNA polymerase (NEB, Cat#M0530L) to PCR amplify SELEX libraries. Other high-fidelity DNA polymerases can be used for this purpose.

***Alternatives:*** We used Ni Sepharose 6 Fast Flow resin (Cytiva, Cat#17531806) corresponding to nickel-charged agarose beads for the purification of histidine-tagged proteins. If a different affinity tag was used, choose the appropriate reagent (e.g., glutathione resin for the purification of GST-tagged proteins).

***Alternatives:*** Here, we used the MinElute PCR purification kit (Qiagen, Cat#28004). If using an alternative kit, check that the minimum size of purified products is compatible with the purification of SELEX libraries (83 bp).

***Alternatives:*** Here, we used KAPA Pure beads (Roche, Cat#07983271001) to clean-up high-throughput sequencing libraries. Alternative reagents can be used, such as AMPure XP beads (Beckman Coulter, Cat#A63880).

• Oligonucleotides for the generation and amplification of SELEX libraries.

**Table:** Order the following oligonucleotides (see **generation of cycle 0 libraries** and **SELEX protocol**):

Name	Sequence
Random library 1	TACACGACGCTCTTCCGATCTNNNNNNNNNNNNNNNNNNNNAGATCGGAAGAG CACACGTCTG
Random library 2	TACACGACGCTCTTCCGATCTNNNNNNNNNNNNNNNNNNNNAGATCGGAAGAG CACACGTCTG
Random library 3	TACACGACGCTCTTCCGATCTNNNNNNNNNNNNNNNNNNNNAGATCGGAAGAG CACACGTCTG
Library FW	ACACTCTTTCCCTACACGACGCTCTTCCGATCT
Library RV	CTGGAGTTCAGACGTGTGCTCTTCCGATCT

**CRITICAL:** “N” refers to random nucleotides (25%A, 25%T, 25%G, 25%C). It is important to **order oligonucleotides only with standard desalting, and no extra purification step** (e.g., PAGE/HPLC purification) which risks excluding some DNA sequences and biasing the randomness of libraries.

***Note:*** HT-SELEX has been validated with random inserts ranging from 14 bp to 40 bp (Jolma et al., 2010, 2013; Nitta et al., 2015). In this protocol we chose a 20 bp insert, which covers motifs for the vast majority of sequence-specific DNA-binding proteins (i.e., those with a binding site ≤20 bp).

• Oligonucleotides for the generation of high-throughput sequencing libraries.

**Table:** Order the following oligonucleotides (see **generation of HT-SELEX libraries for Illumina sequencing**):

Name	Sequence
Seqlib FW	AATGATACGGCGACCACCGAGATCTACACTCTTTCCCTACACGACGC
Seqlib RV 1	CAAGCAGAAGACGGCATACGAGATCCAAGTCCGTGACTGGAGTTCAGACGTGTGCTCT
Seqlib RV 2	CAAGCAGAAGACGGCATACGAGATCAGTGGATGTGACTGGAGTTCAGACGTGTGCTCT
Seqlib RV 3	CAAGCAGAAGACGGCATACGAGATCTAGCTTGGTGACTGGAGTTCAGACGTGTGCTCT
Seqlib RV 4	CAAGCAGAAGACGGCATACGAGATGAGTCCAAGTGACTGGAGTTCAGACGTGTGCTCT
Seqlib RV 5	CAAGCAGAAGACGGCATACGAGATTCCGGATTGTGACTGGAGTTCAGACGTGTGCTCT
Seqlib RV 6	CAAGCAGAAGACGGCATACGAGATAAGGTACCGTGACTGGAGTTCAGACGTGTGCTCT
Seqlib RV 7	CAAGCAGAAGACGGCATACGAGATGGAACGTTGTGACTGGAGTTCAGACGTGTGCTCT
Seqlib RV 8	CAAGCAGAAGACGGCATACGAGATGGCCTCATGTGACTGGAGTTCAGACGTGTGCTCT
Seqlib RV 9	CAAGCAGAAGACGGCATACGAGATATCTTAGTGTGACTGGAGTTCAGACGTGTGCTCT
Seqlib RV 10	CAAGCAGAAGACGGCATACGAGATCTTCACGGGTGACTGGAGTTCAGACGTGTGCTCT
Seqlib RV 11	CAAGCAGAAGACGGCATACGAGATTCCTGTAAGTGACTGGAGTTCAGACGTGTGCTCT
Seqlib RV 12	CAAGCAGAAGACGGCATACGAGATCCTCGGTAGTGACTGGAGTTCAGACGTGTGCTCT
Seqlib RV 13	CAAGCAGAAGACGGCATACGAGATATGAGGCTGTGACTGGAGTTCAGACGTGTGCTCT
Seqlib RV 14	CAAGCAGAAGACGGCATACGAGATGCAGAATCGTGACTGGAGTTCAGACGTGTGCTCT
Seqlib RV 15	CAAGCAGAAGACGGCATACGAGATTGTCGTAGGTGACTGGAGTTCAGACGTGTGCTCT
Seqlib RV 16	CAAGCAGAAGACGGCATACGAGATTAGAGCGCGTGACTGGAGTTCAGACGTGTGCTCT
Seqlib RV 17	CAAGCAGAAGACGGCATACGAGATGGTTCACCGTGACTGGAGTTCAGACGTGTGCTCT
Seqlib RV 18	CAAGCAGAAGACGGCATACGAGATCATTGTTGGTGACTGGAGTTCAGACGTGTGCTCT
Seqlib RV 19	CAAGCAGAAGACGGCATACGAGATACGCCGCAGTGACTGGAGTTCAGACGTGTGCTCT
Seqlib RV 20	CAAGCAGAAGACGGCATACGAGATGTATTATGGTGACTGGAGTTCAGACGTGTGCTCT
Seqlib RV 21	CAAGCAGAAGACGGCATACGAGATAGCGAGCTGTGACTGGAGTTCAGACGTGTGCTCT

***Note:*** Each “Seqlib RV” primer contains a unique 8 bp barcode (underlined) which will be used to tag HT-SELEX samples. This will allow the pooling of multiple libraries for high-throughput sequencing and their subsequent de-multiplexing. If designing additional “Seqlib RV” primers, make sure that all barcodes contain at least two mismatches between each other, and that the base composition of barcodes is homogenous at every position.

• Preparation of buffers for the HT-SELEX protocol.

**Table:** SELEX Buffer (10 mM Tris-HCl pH7.5, 50 mM NaCl, 1 mM MgCl2, 0.5 mM EDTA, 4% Glycerol)

Reagent	Final concentration	Amount
1 M Tris-HCl pH7.5	10 mM	10 mL
5 M NaCl	50 mM	10 mL
1 M MgCl_2_	1 mM	1 mL
0.5 M EDTA	0.5 mM	1 mL
Glycerol	4% (v/v)	40 mL
H_2_O		up to 1 L
**Total**	**n/a**	**1 L**

Store at 4°C for up to 6 months.

**Table:** 1 mg/mL Poly(deoxyinosinic-deoxycytidylic) acid sodium salt (poly(dI-dC)) solution

Reagent	Final concentration	Amount
Poly(dI-dC)	1 mg/mL	10 units
H_2_O		= 10,000 / molecular mass (in units/mg) μL
**Total**	**1 mg/mL**	**Variable (≈800 μL)**

Store aliquots at −20°C.

***Note:*** The molecular mass of poly(dI-dC) (Merck Life Science, Cat#P4929) is lot-dependent. Calculate the precise amount of water to add each time.

**Table:** 1 M DTT solution

Reagent	Final concentration	Amount
DTT	1 M	154.25 mg
H_2_O		up to 1 mL
**Total**	**1 M**	**1 mL**

Store aliquots at −20°C.

• Preparation of 10% polyacrylamide gels for electrophoresis.

**Table:** 10% polyacrylamide solution (enough for 6× gels)

Reagent	Final concentration	Amount
30% Acrylamide/Bis Solution	10%	10 mL
10× TBE buffer	1×	3 mL
H_2_O	n/a	17 mL
100 mg/mL Ammonium persulfate solution	1 mg/mL	300 μL
TEMED	0.1%	30 μL
**Total**	**n/a**	**30 mL**

**CRITICAL:** Add Ammonium persulfate and Tetramethylethylenediamine (TEMED) last to induce polymerization. Quickly cast gels following the addition of these reagents.

## Step-by-step method details

### Perform SELEX (repeat these steps 2–6 times)

**Timing: 1.5 days (×2–6)**

During SELEX, a library of random oligonucleotides is mixed with a DNA-binding protein of interest fused with an affinity tag. Protein-DNA complexes are purified and bound sequences are amplified by the polymerase chain reaction (PCR). This material is re-used for successive cycles of SELEX until most of the library contains high affinity binding sites. For transcription factors, 2–3 cycles are usually sufficient for successful HT-SELEX (Jolma et al., 2010, 2013). However, we performed up to 6× SELEX cycles to characterize SALL4 ZFC4 which promiscuously binds to multiple AT-rich sequences (Pantier et al., 2021).

1. Prepare buffers.

On the day of the experiment, prepare a mastermix of “SELEX binding buffer” (SELEX buffer supplemented with 5 μg/mL poly(dI-dC) and 0.5 mM DTT) and “SELEX wash buffer” (SELEX buffer supplemented with 0.5 mM DTT).

**Table:** SELEX binding buffer (for up to 500 μL of Ni Sepharose 6 Fast Flow resin)

Reagent	Final concentration	Amount
SELEX Buffer	n/a	5 mL
1 M DTT	0.5 mM	2.5 μL
1 mg/mL poly(dI-dC)	5 μg/mL	25 μL
**Total**	**n/a**	**5 mL**

Keep on ice until use.

**Table:** SELEX wash buffer (for N samples)

Reagent	Final concentration	Amount
SELEX Buffer	n/a	N × 6 mL
1 M DTT	0.5 mM	N × 3 μL
**Total**	**n/a**	**n/a**

Keep on ice until use.

2. Equilibrate Ni Sepharose 6 Fast Flow beads in SELEX binding buffer.
  a. Take out the required amount of Ni Sepharose 6 Fast Flow resin (55 μL × number of samples) and transfer into a in a 1.5 mL tube (e.g., for 6 samples, take out 330 μL of Ni Sepharose 6 Fast Flow resin).
    ***Note:*** The total amount of resin includes a 10% excess to account for small inaccuracies when pipetting multiple samples.
    ***Note:*** If a large volume of Ni Sepharose 6 Fast Flow resin is required, split into several 1.5 mL tubes (maximum 500 μL resin/tube) and prepare additional SELEX binding buffer accordingly.
  b. Add 1 mL of SELEX binding buffer and resuspend beads thoroughly by inverting the tube multiple times.
  c. Centrifuge for 1 min at 400 × *g*. Discard the supernatant without disturbing the beads pellet.
  d. Wash beads 2× more times (steps 2b-c).
  e. Resuspend beads in SELEX binding buffer in the initial volume of resin pipetted in step a (e.g., for 6 samples, resuspend in a total volume of 330 μL). Keep on ice until use.
3. Incubate DNA-binding proteins with SELEX libraries.
  a. Set up SELEX reactions in 1.5 mL tubes:

**Table:** SELEX reaction

Reagent	Final concentration	Amount
Histidine-tagged DNA-binding protein	10 μg/mL	1 μg
SELEX DNA library (cycle N-1)	1 μg/mL (15 μg/mL for the first cycle)	200 ng (1.5 μg for the first cycle)
SELEX binding buffer	n/a	up to 100 μL
**Total**	**n/a**	**100 μL**

    ***Note:*** For the first SELEX cycle, use 1.5 μg of “cycle 0” random library (**see generation of cycle 0 libraries**). For subsequent cycles, use 200 ng of SELEX library from the previous cycle (e.g., To perform SELEX cycle 2, use library amplified at the end of cycle 1).
    ***Note:*** It is important to include a negative control SELEX reaction, without addition of proteins, to control for any sequence bias that could be associated with repeated PCR cycling. It is also advised to perform SELEX with independent libraries, which are used as technical replicates (**see generation of cycle 0 libraries**).
    e.g., Sample 1: SALL4 ZFC4 + library 1 (replicate 1).
     Sample 2: SALL4 ZFC4 + library 2 (replicate 2).
     Sample 3: SALL4 ZFC4 + library 3 (replicate 3).
     Sample 4: Negative control (no protein) + library 1 (replicate 1).
     Sample 5: Negative control (no protein) + library 2 (replicate 2).
     Sample 6: Negative control (no protein) + library 3 (replicate 3).
  b. Incubate on a rotating wheel for 10 min at room temperature.
4. Purify protein-DNA complexes.
  a. To capture protein-DNA complexes, add 50 μL of equilibrated Ni Sepharose 6 Fast Flow resin (from step 2) to each SELEX sample.
  b. Incubate for 20 min on a rotating wheel at room temperature.
  c. To remove non-specifically bound DNA-protein complexes, add 1 mL of SELEX wash buffer and resuspend beads thoroughly by inverting the tube multiple times.
  d. Centrifuge for 1 min at 400 × *g*. Discard the supernatant without disturbing the beads pellet.
  e. Wash beads 4× more times (steps 4c-d).
  f. Resuspend the resin in 100 μL H_2_O.

***Note:*** Elution of DNA from the beads is not necessary, as this material can be directly used as a template for PCR amplification of SELEX libraries.

**Pause point:** The resin (protein-DNA complexes) can be stored at −20°C (long term). This material can be used at a later time for PCR amplification.

5. PCR-amplify enriched DNA.
  **CRITICAL:** The amount of DNA bound to the resin is unknown and usually varies between SELEX samples. Therefore, it is important to empirically determine the optimal number of PCR cycles to amplify each SELEX library (see the following steps).
  a. For each SELEX sample, prepare a PCR mastermix (for 4× PCR reactions) in a 1.5 mL tube:

**Table:** PCR reaction master mix

Reagent	Amount
**Protein-DNA complexes (bead suspension)**	22.5 μL
5× Phusion HF Buffer	45 μL
dNTPs (10 mM)	4.5 μL
Library FW (10 μM)	11.25 μL
Library RV (10 μM)	11.25 μL
Phusion DNA Polymerase	2.25 μL
Nuclease-free water	128.25 μL
**Total**	**225 μL**

    ***Note:*** It is not recommended to increase the amount of resin (DNA template) in the mix, as an excess can inhibit the PCR reaction.
  b. Divide mastermix between 4 PCR tubes (50 μL/tube).
    ***Note:*** Before transferring the mix to PCR tubes, ensure that beads are homogeneously resuspended by pipetting up and down multiple times.
  c. Run each of the 4× PCR reactions with a different PCR programme (increasing numbers of PCR cycles):

**Table:** PCR cycling conditions (8× cycles)

Steps	Temperature	Time	Cycles
Initial Denaturation	98°C	1 min	1
Denaturation	98°C	20 s	**8 cycles**
Annealing	68°C	20 s	
Extension	72°C	20 s	
Final extension	72°C	5 min	1
Hold	4°C	forever

**Table:** PCR cycling conditions (12× cycles)

Steps	Temperature	Time	Cycles
Initial Denaturation	98°C	1 min	1
Denaturation	98°C	20 s	**12 cycles**
Annealing	68°C	20 s	
Extension	72°C	20 s	
Final extension	72°C	5 min	1
Hold	4°C	forever

**Table:** PCR cycling conditions (15× cycles)

Steps	Temperature	Time	Cycles
Initial Denaturation	98°C	1 min	1
Denaturation	98°C	20 s	**15 cycles**
Annealing	68°C	20 s	
Extension	72°C	20 s	
Final extension	72°C	5 min	1
Hold	4°C	forever

**Table:** PCR cycling conditions (20× cycles)

Steps	Temperature	Time	Cycles
Initial Denaturation	98°C	1 min	1
Denaturation	98°C	20 s	**20 cycles**
Annealing	68°C	20 s	
Extension	72°C	20 s	
Final extension	72°C	5 min	1
Hold	4°C	forever
  d. To control the amplification of libraries, run a small amount of PCR reaction (5 μL) on a 10% polyacrylamide gel and stain with a 0.5 μg/mL ethidium bromide solution (see Figure 3).

**Figure 3:**
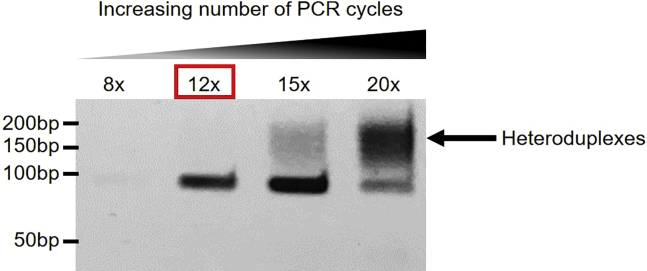
10% polyacrylamide gel showing the optimization of PCR conditions to amplify enriched DNA from Ni Sepharose beads following a cycle of SELEX For this sample, we purified the product following to 12× PCR cycles, as it showed a robust amplification of the library (83 bp) without detectable heteroduplexes (see troubleshooting 1).
  e. For each SELEX sample, select the optimal PCR reaction and discard other tubes (see Figure 3).
  f. Purify DNA using the Qiagen MinElute PCR purification kit and following manufacturer’s protocol. Elute with 20 μL of EB Buffer (included in the kit, 10 mM Tris-HCl pH8.5) or H_2_O.
    ***Note:*** A single PCR reaction will yield enough DNA to proceed with the protocol.
  g. Evaluate DNA concentration and integrity of purified SELEX libraries using a Nanodrop spectrophotometer.
    **Pause point:** Store purified SELEX libraries at −20°C (long term).
  h. Use DNA as an input to repeat an additional cycle of SELEX (N+1).
    **CRITICAL:** Remember to save an aliquot of purified SELEX library (≈20 ng) for high-throughput sequencing (see **generation of HT-SELEX libraries for Illumina sequencing**).

### Generate HT-SELEX libraries for Illumina sequencing

**Timing: 1.5 days**

**Figure 4:**
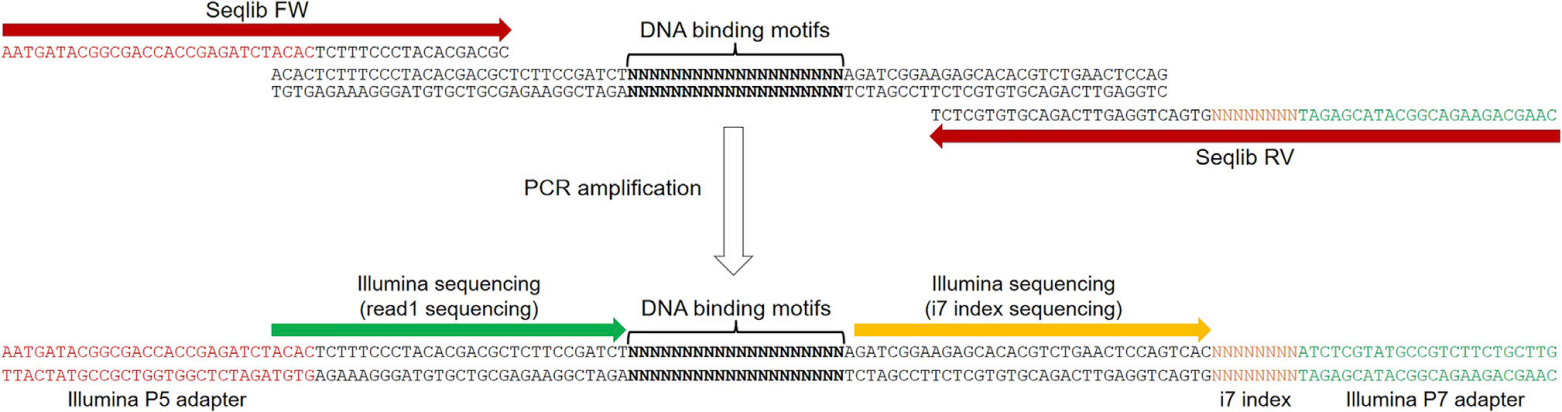
Strategy for generating HT-SELEX libraries for high-throughput sequencing Following PCR, each SELEX library contains Illumina sequencing adapters (P5/P7) and unique barcodes (i7 indexes).

After multiple SELEX cycles, DNA libraries contain a significant proportion of high affinity DNA binding sites for the target protein. This step describes the conversion of SELEX libraries into HT-SELEX libraries containing Illumina adapters and unique barcodes (see Figure 4). These samples are subsequently pooled and submitted to high-throughput sequencing to reveal preferred DNA motifs.

6. Select SELEX samples to submit to high-throughput sequencing.

***Note:*** For most purposes, it is not necessary to sequence libraries for all SELEX cycles. However, it is important to sequence initial random libraries (cycle 0) in order to assess the initial frequency of all DNA motifs. While libraries at the last SELEX cycle will contain the highest proportion of DNA binding motifs, sequencing intermediate SELEX cycles will provide useful information regarding the kinetics of enrichment of preferred DNA motifs.

**CRITICAL:** For all selected SELEX cycles, include technical replicates (i.e., different libraries) as well as a negative control (i.e., without addition of DNA-binding proteins). These important controls will allow the measurement of technical bias during the SELEX protocol (e.g., base composition bias of DNA polymerase during PCR amplification).

Here is an example of SELEX dataset for SALL4 ZFC4 (6× SELEX cycles, 3 replicates):

 Sample 1: Cycle0 - Initial random SELEX library 1 (replicate 1).

 Sample 2: Cycle0 - Initial random SELEX library 2 (replicate 2).

 Sample 3: Cycle0 - Initial random SELEX library 3 (replicate 3).

 Sample 4: Cycle 1 - SALL4 ZFC4 (replicate 1).

 Sample 5: Cycle 1 - SALL4 ZFC4 (replicate 2).

 Sample 6: Cycle 1 - SALL4 ZFC4 (replicate 3).

 Sample 7: Cycle 1 - Negative control (no protein) (replicate 1).

 Sample 8: Cycle 1 - Negative control (no protein) (replicate 2).

 Sample 9: Cycle 1 - Negative control (no protein) (replicate 3).

 Sample 10: Cycle 3 - SALL4 ZFC4 (replicate 1).

 Sample 11: Cycle 3 - SALL4 ZFC4 (replicate 2).

 Sample 12: Cycle 3 - SALL4 ZFC4 (replicate 3).

 Sample 13: Cycle 3 - Negative control (no protein) (replicate 1).

 Sample 14: Cycle 3 - Negative control (no protein) (replicate 2).

 Sample 15: Cycle 3 - Negative control (no protein) (replicate 3).

 Sample 16: Cycle 6 - SALL4 ZFC4 (replicate 1).

 Sample 17: Cycle 6 - SALL4 ZFC4 (replicate 2).

 Sample 18: Cycle 6 - SALL4 ZFC4 (replicate 3).

 Sample 19: Cycle 6 - Negative control (no protein) (replicate 1).

 Sample 20: Cycle 6 - Negative control (no protein) (replicate 2).

 Sample 21: Cycle 6 - Negative control (no protein) (replicate 3).

7. PCR amplify HT-SELEX libraries.
  **CRITICAL:** PCR conditions were optimized to amplify HT-SELEX libraries. However, the amount of DNA template and the number of PCR cycles might need to be adjusted in order to avoid the formation of heteroduplexes (see troubleshooting 1).
  a. For each SELEX sample, set a PCR reaction in a PCR tube **using a unique reverse primer (Seqlib RV)**:

**Table:** PCR reaction mix

Reagent	Amount
SELEX library DNA template	20 ng **Might be adjusted**
5× Phusion HF Buffer	10 μL
dNTPs (10 mM)	1 μL
Seqlib FW (10 μM)	2.5 μL
Seqlib RV (10 μM) - **unique for each sample**	2.5 μL
Phusion DNA Polymerase	0.5 μL
Nuclease-free water	up to 50 μL
**Total**	**50 μL**

    ***Note:*** Each “Seqlib RV” primer contains a distinct barcode which will allow the pooling of multiple samples for high-throughput sequencing (see materials and equipment).
  b. Run the following PCR programme:

**Table:** PCR cycling conditions

Steps	Temperature	Time	Cycles
Initial Denaturation	98°C	1 min	1
Denaturation	98°C	20 s	1 (initial PCR step)
Annealing	60°C	20 s	
Extension	72°C	20 s	
Denaturation	98°C	20 s	4 cycles
Annealing	68°C	20 s	**Might be adjusted**
Extension	72°C	20 s	
Final extension	72°C	5 min	1
Hold	4°C	forever
  c. To control the amplification of libraries, run a small amount of PCR reaction (5 μL) on a 10% polyacrylamide gel and stain with a 0.5 μg/mL ethidium bromide solution (see Figure 5).

**Figure 5:**
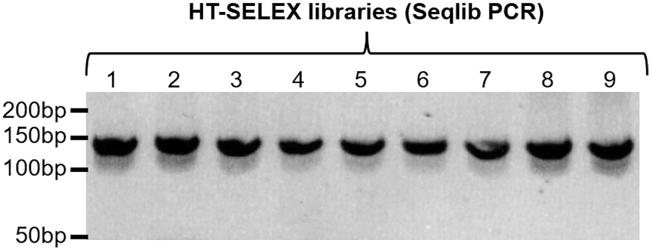
10% polyacrylamide gel showing the generation of 9× HT-SELEX libraries (144 bp) for high-throughput sequencing
  d. Purify HT-SELEX libraries using the Qiagen MinElute PCR purification kit and following manufacturer’s protocol. Elute with 20 μL of EB Buffer (included in the kit, 10 mM Tris-HCl pH8.5) or H_2_O.
    ***Note:*** For each SELEX sample, a single PCR reaction will yield enough DNA to proceed with high-throughput sequencing.
    ***Note:*** Long PCR primers were used to generate HT-SELEX libraries, and these oligonucleotides are not completely eliminated following PCR purification with the Qiagen MinElute column.
  e. Evaluate DNA concentration and integrity of purified HT-SELEX libraries using a Nanodrop spectrophotometer.
    **Pause point:** Store purified HT-SELEX libraries at −20°C (long term). These samples can be pooled and submitted to high-throughput sequencing at a later time.

**Figure 6:**
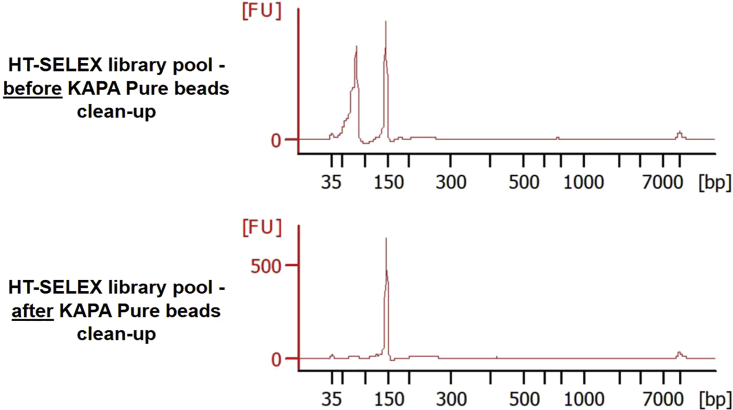
Bioanalyzer profile showing the successful elimination of PCR primers from HT-SELEX library pool following a 3× clean-up with KAPA Pure beads
  c. Perform a final quality control on the library pool using the Agilent High Sensitivity DNA Kit and the 2100 Bioanalyzer instrument (following manufacturer’s protocol) (see Figure 6).
    ***Alternatives:*** Run the library pool on a 10% polyacrylamide gel and stain with a 0.5 μg/mL ethidium bromide solution, as previously described.
  d. Submit the HT-SELEX library pool to high-throughput sequencing using an Illumina instrument (e.g., Miseq/NextSeq/NovaSeq). Single-end sequencing is sufficient to cover the 20 bp insert containing putative DNA binding motifs (see Figure 4). A sequencing depth of 10,000–50,000 reads per sample should be sufficient to obtain robust quantification of DNA motifs for HT-SELEX (see troubleshooting 2).

8. Prepare a sequencing library pool and submit to high-throughput sequencing.
  a. Use Nanodrop quantification to pool all HT-SELEX libraries in equimolar amounts in a 1.5 mL tube.
    **CRITICAL:** Make sure that all libraries in the pool contain unique indexes, so that each library can be de-multiplexed following high-throughput sequencing.
  b. To ensure complete removal of leftover PCR primers contaminating libraries, perform a clean-up with KAPA Pure beads following manufacturer’s protocol. **Use a 3× bead-to-sample ratio** (e.g., add 150 μL of beads to 50 μL of HT-SELEX pool) to eliminate oligonucleotides below 100 bp (see Figure 6).
    **Pause point:** Store purified HT-SELEX library pool at −20°C (long term). This material can be submitted to high-throughput sequencing at a later time.

## Expected outcomes

The final output of the HT-SELEX protocol is the library pool subjected to Illumina sequencing (see Figure 6). Intermediate material corresponding to protein-DNA complexes (bead suspension) and purified SELEX libraries without Illumina adapters can be stored long term at −20°C (**see Pause steps during the SELEX protocol**).

The section below describes a complete bioinformatic workflow to process sequencing data and quantify the enrichment of DNA motifs. SALL4 ZFC4 HT-SELEX dataset (including processed files) is available in ArrayExpress: E-MTAB-9236. Additionally, we sequenced the same libraries at higher throughput to determine the minimal sequencing depth for HT-SELEX analysis (see troubleshooting 2). This new dataset is also available in ArrayExpress: E-MTAB-11484.

## Quantification and statistical analysis

### Bioinformatic analysis

**Timing: 1 day**

***Note:*** Analysis time will vary depending on the sequencing depth of HT-SELEX datasets and the length of DNA motifs (k-mers) to analyze.

1. Setup the package management system “conda” following the instructions available here: https://docs.conda.io/projects/conda/en/latest/user-guide/install/index.html.
2. Install all required software inside a conda environment from your command line:

> conda create -n eme_selex -c bioconda flexbar snakemake pip jupyterlab tqdm pandas seaborn

> conda activate eme_selex

> pip install eme_selex logomaker upsetplot

> conda install -c plotly plotly==5.6.0

3. Generate a tab-separated values (TSV) file containing metadata of your HT-SELEX samples using the following format:

Samplename  library protein cycle

RV## lib# None 0

RV## lib# None 0

RV## lib# None 0

RV## lib# ZFC4 6

RV## lib# ZFC4 6

RV## lib# ZFC4 6

4. Pre-process and quality-trim sequencing reads.
  **CRITICAL:** Trim sequencing reads to the exact size of the library insert (in our case 20 bp). For more information, regarding library design, see materials and equipment section.
  a. Execute flexbar for each individual sample using the following parameters:
    > flexbar --reads {input} --post-trim-length **20** --min-read-length 20 --qtrim-threshold 30 --output-reads {output} –fasta-output
    --number-tags --stdout-log > {log}
    ***Note:*** Use a workflow manager such as Snakemake (https://snakemake.readthedocs.io) to automate this step for all samples.

5. Calculate k-mer frequency using the Python package eme_selex (tested on python version 3.10).
  a. Calculate the abundance of 5-mer motifs for all samples using the following Python code:
    from collections import defaultdict
    from eme_selex.eme import kmer_fraction_from_file as kf
    counts, fractions, models = defaultdict(dict), defaultdict(dict), \ defaultdict(dict)
    samples = [f"RV{s:02d}" for s in range(1, 22)]
    k = 5
    for sample in df["SampleName"].values:
     c, f, m = kf(f"fasta/{sample}.fasta.gz", k=k)
     counts[sample] = c
     fractions[sample] = f
     models[sample] = m
    ***Note:*** The choice of k-mers length (up to 10 bp, see limitations) depends on the DNA-binding protein of interest. In our case, we determined that SALL4 binds to short DNA motifs of 3–5 bp (Pantier et al., 2021).
  b. Normalize the data and generate a data frame containing fold-change (vs cycle 0) values. Please refer to our bioinformatic workflow documentation (https://eme-selex.readthedocs.io) for source code.
6. Visualize the enrichment of DNA motifs following HT-SELEX.

**Figure 7:**
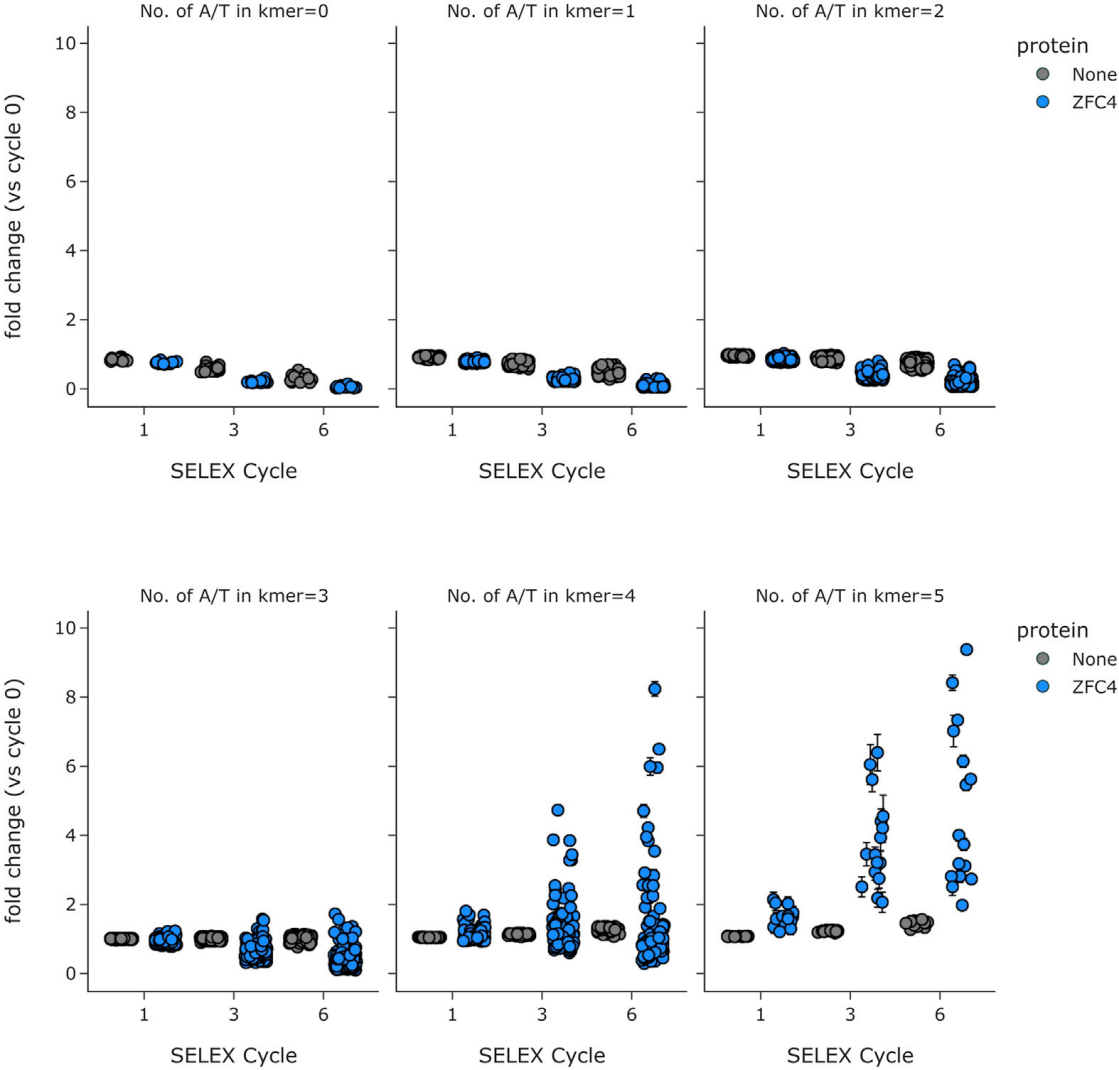
Enrichment of all possible 5-mer DNA motifs during the HT-SELEX protocol for SALL4 ZFC4 (blue) compared to negative control (gray) DNA motifs (k-mers) were divided into six categories of increasing A/T content. Error bars indicate the technical variability with independent SELEX libraries.

***Note:*** To observe DNA binding of ZFC4 according to DNA base composition, we divided all 5-mer motifs into different categories depending on their proportion of A/T nucleotides (see Figure 7). Please refer to our bioinformatic workflow documentation (https://eme-selex.readthedocs.io) for source code.

**CRITICAL:** Always compare the enrichment of k-mers (DNA motifs) with the initial random library (cycle 0) and negative control (see Figure 7). These controls will confirm the specific enrichment of DNA motifs during the SELEX protocol.

***Note:*** Here, we observed a progressive enrichment of a large number of AT-rich k-mers throughout the SELEX protocol (cycles 1/3/6), which confirmed promiscuous binding of SALL4 ZFC4. In the case of specific DNA binding, only few DNA motifs would have been enriched, with high similarity to the most abundant k-mer (Jolma et al., 2010).

## Limitations

HT-SELEX relies on the detection of protein-DNA interactions *in vitro*. Alternative HT-SELEX protocols were developed to study binding to other substrates such as methylated DNA (Yin et al., 2017) and RNA (Jolma et al., 2020). However, this technique is not suitable for proteins binding indirectly to DNA, for example via interactions with histones or via protein-protein interactions with transcription factors.

It is often necessary to express small protein fragments (e.g., C2H2 zinc-fingers, Homeodomain) rather than full-length proteins. However, this strategy is not possible for proteins for which the DNA-binding domain has not yet been mapped.

Our Python package “eme_selex” is developed to analyze and quantify the abundance of k-mers up to 10 bp, which is sufficient for most transcription factors. Analyzing k-mers of length 11 bp or higher is computationally challenging for a personal computer, and is therefore not possible at this point using eme_selex.

## Troubleshooting

### Problem 1

How to determine optimal PCR conditions to amplify SELEX libraries.

**Figure 8:**
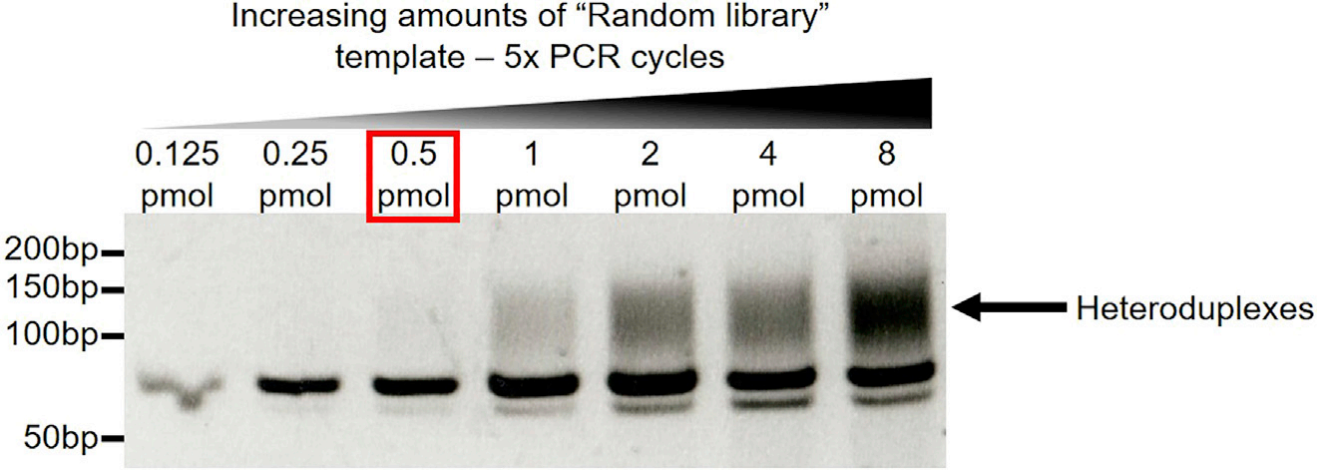
10% polyacrylamide gel showing the optimization of the amount of DNA template to generate cycle 0 SELEX libraries 0.5 pmol of “Random library” per 50 μL PCR reaction (10 nM concentration) results in robust amplification of the library after 5× PCR cycles without detectable heteroduplexes.

Over-amplification or excessive amounts of DNA template will result in the formation of heteroduplexes (also known as “bubble products”) due to annealing of mismatched sequences (Thompson et al., 2002; Kanagawa, 2003). These unwanted products containing secondary structures can be detected by gel electrophoresis, as they run higher than their expected size (see Figures 3 and 8).

### Potential solution

To determine optimal PCR conditions to amplify SELEX libraries **(see generation of cycle 0 libraries and SELEX protocol**), two strategies can be adopted:

Perform the same PCR multiple times with increasing amounts of DNA template and a fixed number of PCR cycles (see Figure 8).

Alternatively, perform the same PCR multiple times with a fixed amount of DNA template and increasing numbers of PCR cycles (see Figure 3).

### Problem 2

How to determine the optimal sequencing depth for HT-SELEX analysis.

### Potential solution

**Figure 9:**
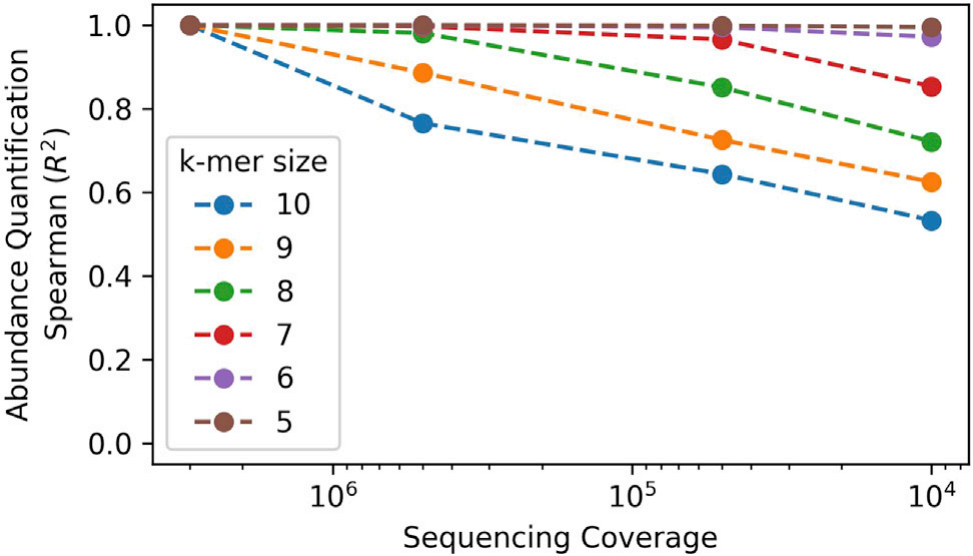
Rank comparison (Spearman R^2^) between k-mer abundance in sub-sampled libraries compared with original samples (≈3,000,000 reads per sample)

In our previous study (Pantier et al., 2021), we sequenced SALL4 ZFC4 HT-SELEX libraries with an average sequencing depth of 20,000 reads per sample (ArrayExpress: E-MTAB-9236). In order to determine the optimal sequencing depth for HT-SELEX analysis (**see generation of HT-SELEX libraries for Illumina sequencing**), we re-sequenced the same libraries with a very high coverage of ≈3,000,000 reads per sample (ArrayExpress: E-MTAB-11484). Using this new dataset, we simulated varying coverages by sub-sampling 500,000, 50,000 and 10,000 reads, respectively. For all conditions, we calculated the abundance of all k-mers (from 5 to 10 bp) using eme_selex, and compared their ranks with the highest coverage dataset (see Figure 9). We found a very high correlation (Spearman R^2^) between samples at all sequencing depths for short DNA motifs (k-mers length 5–6 bp), corresponding to ZFC4 binding sites. These results indicate that accurate quantification of k-mer abundance can still be obtained at low sequencing coverage (≈10,000 reads per sample). Higher sequencing coverage (at least 500,000 reads per sample) would be recommended to investigate promiscuous binding to long DNA motifs (k-mers length >7 bp).

***Note:*** The number of DNA motifs increases exponentially when *k* increases from to 5 to 10 bp. Comparing the abundance of k-mers across varying sequencing depth is meaningful only for proteins binding promiscuously to a large number of DNA motifs. For more information regarding the overlap of top-ranking DNA motifs, please refer to our bioinformatic workflow documentation: https://eme-selex.readthedocs.io/en/latest/coverage.html.

## Resource availability

### Lead contact

Further information and requests for resources and reagents should be directed to and will be fulfilled by the lead contact, Adrian Bird (a.bird@ed.ac.uk).

### Materials availability

Reagents generated in this study (expression plasmids, histidine tagged proteins) are available upon request.

## Data Availability

HT-SELEX datasets (including processed files) are available in ArrayExpress: E-MTAB-9236, E-MTAB-11484. eme_selex is available on GitHub: https://github.com/kashyapchhatbar/eme_selex (https://doi.org/10.5281/zenodo.6586738). Complete documentation is available here: https://eme-selex.readthedocs.io.
